# Tangeretin Unravels Metabolic Dysfunction-Associated Fatty Liver Disease in Rats by Enhancing the IRS/Akt Pathway

**DOI:** 10.3390/life15030491

**Published:** 2025-03-18

**Authors:** Ittipon Yuenyong, Prapassorn Potue, Putcharawipa Maneesai, Juthamas Khamseekaew, Apiwan Manimmanakorn, Metee Iampanichakul, Poungrat Pakdeechote

**Affiliations:** Department of Physiology, Faculty of Medicine, Khon Kaen University, Khon Kaen 40002, Thailand; bankyuenyong@kkumail.com (I.Y.); prappo@kku.ac.th (P.P.); putcma@kku.ac.th (P.M.); juthakh@kku.ac.th (J.K.); mapiwa@kku.ac.th (A.M.); meteiam@kku.ac.th (M.I.)

**Keywords:** tangeretin, MAFLD, high-fat diet, insulin resistance, oxidative stress

## Abstract

Excessive high-fat diet (HFD) intake can precipitate metabolic dysfunction-associated fatty liver disease (MAFLD). Tangeretin is a citrus flavonoid possessing many biological properties. We examined the impact of tangeretin on MAFLD and its underlying mechanism. Rats were given HFD plus 15% fructose solution for four months to produce metabolic syndrome. Metabolic syndrome rats were administered 100 mg/kg of metformin or 25 mg/kg of tangeretin for the last four weeks. HFD-induced increased body weight, liver weight, adipose tissue weight, fasting blood glucose, serum insulin, total triglyceride, total cholesterol, and free fatty acids and reduced adiponectin and high-density lipoprotein cholesterol levels in metabolic syndrome, which were alleviated by tangeretin (*p* < 0.05). Tangeretin stabilized alanine transaminase activity, liver catalase, and inflammatory and oxidative stress markers in HFD rats compared to untreated HFD rats (*p* < 0.05). Tangeretin reduced hepatic steatosis induced by HFD. Downregulation of hepatic insulin receptor substrate-1 (IRS-1) and protein kinase B (Akt) protein expression in metabolic syndrome rats was recovered by tangeretin (*p* < 0.05). Metformin, an antihyperglycemic drug, produced comparable effects to tangeretin. In conclusion, tangeretin attenuates metabolic disorders and fatty liver induced by HFD in rats. The underlying mechanisms involve reducing oxidative stress, and inflammation and enhancing insulin sensitivity.

## 1. Introduction

Metabolic syndrome is characterized by a group of metabolic disorders, including insulin resistance, hypertension, dyslipidemia, and central obesity. Metabolic syndrome elevates the risk of further chronic non-communicable diseases, such as type 2 diabetes, cardiovascular disease, and fatty liver disease [1]. Metabolic dysfunction-associated fatty liver disease (MAFLD), a recent name for non-alcoholic fatty liver (NAFLD), is a crucial cause of liver abnormalities [2]. MAFLD criteria include evidence of hepatic steatosis with any of the following three criteria: overweight/obesity, type 2 diabetes, or signs of metabolic dysregulation [3]. It has been well known that MAFLD is a silent chronic disease. Most patients have no symptoms until cirrhosis has developed [4]. MAFLD formation and progression are influenced by multiple factors, including genetics, environment, and lifestyle, notably the excessive intake of high-energy meals [2]. A previous publication demonstrated that animals fed a high-fat diet (HFD) along with a fructose drink developed obesity, insulin resistance, and dyslipidemia [5]. Recently, HFD-induced MAFLD in rats has been characterized by metabolic disorders and oxidative stress [6].

It has been suggested that insulin resistance is found in NAFLD patients and is regarded as a primary contributor to the development of NAFLD and the advancement of liver diseases [3]. When adipose tissue develops insulin resistance and can no longer store additional fat, elevated circulating free fatty acids (FFAs) may result in ectopic fat deposition in the liver and subsequent hepatic insulin resistance [7]. Adiponectin is an adipokine protein that is produced and released by adipose tissue to maintain insulin sensitivity. Low adiponectin levels were linked to insulin resistance and inflammation [8,9]. Significant evidence suggests that inflammation, driven by elevated levels of serum tumor necrosis factor-α (TNF-α) and interleukin 6 (IL-6), is a crucial mechanism in high-fat diet-induced fatty liver and determines the severity of the condition [10,11]. Moreover, accumulating evidence shows that insulin resistance is linked to increased liver tissue damage and may serve as a potential mechanism for the start and progression of non-alcoholic fatty liver disease in obese patients [12]. Under physiological conditions, the major function of insulin in hepatic tissues is to control the metabolism of glucose and lipids via the insulin receptor, phosphoinositide 3-kinase (PI3K), and serine/threonine kinase (Akt) signaling pathway [13]. Recently, evidence has shown the association between hepatic insensitivity and metabolic dysfunction-associated steatosis liver disease induced by an HFD in rats [14]. Another factor implicated in the pathogenesis and progression of MAFLD is oxidative stress since insulin resistance, hyperglycemia, and lipid overload can enhance oxidative stress [15,16]. Animal models of MAFLD/NAFLD show low activities of antioxidant enzymes and high levels of reactive oxygen species [6].

**Figure 1:**
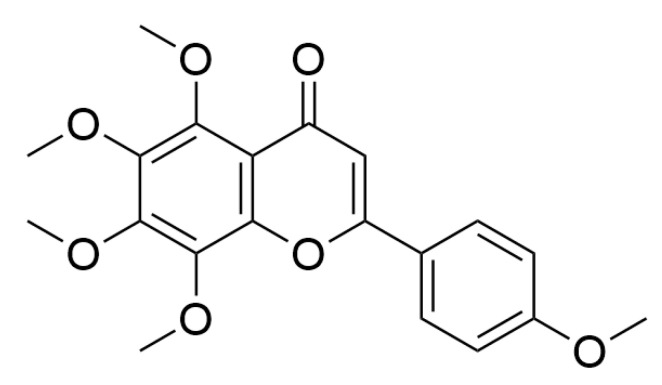
Chemical structure of tangeretin.

Managing MAFLD generally involves lifestyle modifications, medical intervention, and managing hidden risk factors [17]. Moreover, antioxidant substances have been recommended as a possible management option for MAFLD [16]. Tangeretin is a flavonoid, specifically categorized as a polymethoxyflavone. It is frequently present in citrus fruits, including tangerines, oranges, and grapefruits [18]. Figure 1 depicts the chemical structure of tangeretin, which specifically contains five methoxy (CH_3_O) groups attached at well-defined positions on the flavone skeleton. It has been reported that tangeretin has wide properties, such as anti-inflammation, anti-cancer, and antioxidation [19]. The antioxidant properties of tangeretin are largely mediated by the increased expression of nuclear factor erythroid 2-related factor 2 (Nrf2) protein [20]. Tangeretin prevents cardiac injury induced by isoproterenol by alleviating cardiac infarct size linked to decreasing oxidative stress and inflammatory biomarkers and PI3K/Akt protein expression [21]. Tangeretin enhances cell viability and insulin secretion in streptozotocin-induced rat insulinoma cells via its antioxidant and modulates the nuclear factor kappa B (NF-κB) pathway in cell lines [22]. Furthermore, tangeretin alters systemic inflammation, increases fat browning, and impacts gut microbiota to prevent obesity in high-fat diet-induced obese C57BL/6 mice [15]. However, the effects of tangeretin on MAFLD and insulin signaling pathways in liver tissue are still limited. Therefore, this study was designed to investigate the effects of tangeretin on fatty liver parameters, oxidative stress and inflammatory markers, and insulin signaling pathways in rats with HFD-induced MAFLD.

## 2. Materials and Methods

### 2.1. Chemicals

Tangeretin was supplied by ChemFaces Biochemical Co., Ltd. (Wuhan, China), with a specified purity of 98%. Metformin was supplied by Siam Pharmaceutical Company Ltd. (Bangkok, Thailand).

### 2.2. Animal Induction and Experimental Designs

Healthy male Sprague–Dawley rats (body weight 220–250 g) were used in the study and were supplied by Nomura Siam International Co., Ltd., Bangkok, Thailand. The rats were housed in plastic cages (3 rats/cage) in a controlled environment with a heating, ventilation, and air conditioning (HVAC) system, maintaining a temperature of 22 ± 2 °C, relative humidity of 50 ± 10%, and a 12-h light-dark cycle (light at 6:00 a.m.) at the Northeast Laboratory Animal Center, Khon Kaen University, Khon Kaen, Thailand. All animal procedures were conducted following ethical guidelines for working with animals, controlled, and approved by the Institutional Animal Care and Use Committee of Khon Kaen University (Ethics No IACUC-KKU-38/65, Approval date 21 April 2022). After one week of acclimatization to the experimental conditions, all animals were randomly divided into four groups (n = 6/group): control + vehicle (Control), MS + vehicle (MS), MS + 25 mg tangeretin (T25), and MS + 100 mg metformin (Met100).

The control group received tap water and a standard chow diet, while the MS group received a 15% fructose solution and HFD. The standard rat chow diet (/100 g) contained 5.72 g fat, 22.9 g protein, and 57.81 g carbohydrates, whereas an HFD (/100 g) comprised 24.29 g fat, 13.25 g protein, and 46.3 g carbohydrates [23,24]. After 12 weeks of the experimental period, rats fed with HFD were subdivided into three groups: MS group, MS rats received either propylene glycol (PG) as a vehicle; T25, MS rats were treated with 25 mg/kg/day of tangeretin; and Met100, MS rats were treated with 100 mg/kg/day of metformin. Tangeretin was dissolved in PG and was orally administered through a gastric tube daily, starting from weeks 12 to 16. Rat body weights were measured once a week throughout the experiment. The food, fructose, and calorie intake were monitored every day. The doses of metformin [23,25] and tangeretin [26,27,28,29] used in this study were based on a preliminary study and previous reports. Preliminary results indicated that the action of tangeretin on signs of metabolic syndrome at doses of 25 and 50 mg/kg (n = 4) did not differ. Therefore, a 25 mg/kg dose of tangeretin was selected for further investigation.

At the end of the experiments, rats were fasted overnight for 12 h. They were anesthetized by intraperitoneal injection of zoletil 1.0 mg/kg. Blood samples were collected from the abdominal aorta to measure fasting blood glucose (FBG), insulin, lipid profiles, liver function, and oxidative stress markers. Visceral fat and epididymal fat were carefully isolated and weighed.

### 2.3. Fasting Blood Glucose and Oral Glucose Tolerance Test (OGTT)

Rats were given a 20% glucose solution orally at a dose of 2 g/kg body weight following an overnight fast. Using a fresh test strip and a blood glucose meter (ACCU-CHEK^®^, Roche Diagnostics GmbH, Mannheim, Germany), blood glucose levels were determined at 0, 30, 60, 120, and 180 min after a tiny drop of blood was drawn. The area under the curve (AUC) for the OGTT was computed. After an overnight fast, blood glucose levels were measured by taking a small drop of blood and using a new test strip with a blood glucose meter (ACCU-CHEK^®^, Roche Diagnostics GmbH, Mannheim, Germany).

### 2.4. Fasting Serum Insulin and HOMA-IR Index Assessment

Fasting serum insulin was analyzed using a commercial ELISA kit from Millipore Corporation (Billerica, MA, USA). The protocol strictly followed the assay kit guidelines. A sandwich ELISA assay uses a pre-coated microtiter plate with a monoclonal antibody specific to rat insulin. The insulin sample is added to the well, where it binds to the capture antibody. After washing, a biotinylated polyclonal antibody, also specific to rat insulin, is added and binds to the captured insulin. The plate is then washed again, followed by the addition of a streptavidin-HRP conjugate that binds to the biotinylated antibody. After washing away any unbound conjugate, a TMB substrate is introduced to produce color. The reaction is halted with acid, and absorbance is measured at 450 nm. The insulin concentration in the sample is determined by comparing its absorbance to a standard curve created with known concentrations of rat insulin. The relative value of the homeostasis model assessment for insulin resistance (HOMA-IR), serving as the insulin resistance index in the rats, was calculated using the following formula: HOMA-IR index = fasting blood glucose (mg/dL) × fasting insulin (µU/mL)/405.

### 2.5. Adiponectin Level Measurement

The plasma adiponectin level was measured using commercial kits. (Reed Biotechnology, Wuhan, China). This ELISA kit uses a sandwich-ELISA method to measure plasma rat ADP/Acrp30 (adiponectin) levels. The assay starts with a microplate coated with a capture antibody specific to Rat ADP/Acrp30, which preferentially binds the target protein in the sample or reference. A biotinylated detection antibody is subsequently added, resulting in an immunological complex with the attached adiponectin. Then, an Avidin-HRP conjugate is added to aid enzymatic amplification. The addition of a TMB substrate causes a chromogenic reaction, resulting in a blue color in wells holding the target analyte. The reaction is ended using a stop solution, which causes the color to change to yellow. The optical density (OD) is measured at 450 nm, and the concentration of adiponectin is interpolated from a standard curve, resulting in a quantitative assessment of the protein in the sample.

### 2.6. Lipid Profile Assessments

Blood samples were drawn from the abdominal aorta, serum was separated, and prepared to measure values of total cholesterol (TC), triglycerides (TG), and high-density lipoprotein cholesterol (HDL-c) with a commercial kit (Human Gesellschaft Fuer Biochemica und Diagnostica mbH, Wiesbaden, Germany). FFAs were detected using a specific kit (ab65341 Free Fatty Acid, Abcam, Waltham, MA, USA).

### 2.7. Serum Alanine Transaminase (AST) and Aspartate Transaminase (ALT) Activity Measurement

Serum ALT and AST activity were determined using commercial kits (Sigma-Aldrich, Saint Louis, MI, USA). In summary, a blood sample is combined with reagents from the test kit, where the AST or ALT enzymes in the blood react with these chemicals through a multi-step process, ultimately producing a colored product at 450 nm. The color intensity corresponds to the enzyme concentration in the blood. The instrument measures this intensity and compares it to a standard to determine the enzyme level in the sample.

### 2.8. Assay of Superoxide (O_2_^•−^) Production

Hepatic tissues were quickly removed and cut into small pieces (20–25 mg wet weight). O_2_^•−^ production in these sections was assessed using the lucigenin-enhanced chemiluminescence method [30]. After removing connective tissue and fat, the liver samples were placed in an oxygenated Krebs-KCl buffer (pH 7.4) and equilibrated at 37 °C for 30 min. Lucigenin (100 µM) was then added to the sample tubes, which were placed in a luminometer (Turner Biosystems, Sunnyvale, CA, USA). Photon counts were averaged over 5 min with readings integrated every 30 s. The liver samples were then dried at 45 °C for 24 h to determine liver dry weight. O_2_^•−^ production in liver tissue was measured in relative light units per minute per milligram of dry tissue weight.

### 2.9. Measurement of Malondialdehyde (MDA) Levels in Plasma and Liver Tissue

A 200 mg hepatic tissue was homogenized in 800 µL of 0.1 M Tris–HCl buffer (pH 7.4) under ice-cold conditions. After centrifugation, the clear supernatant was collected to measure MDA levels. Spectrophotometric analysis of plasma and tissue supernatant was performed for thiobarbituric acid (TBA) reactive substances [31]. Briefly, 150 µL of the sample was treated with a solution containing 10% TCA, 5 mmol/L EDTA, 8% SDS, and 0.5 g/mL butylated hydroxytoluene (BHT). After a 10-min incubation at room temperature, 0.6% TBA was added, and the solution was boiled for 30 min in a water bath. The mixture was then cooled to room temperature and centrifuged at 10,000× *g* for 5 min. The absorbance of the supernatant was measured at 532 nm using a spectrophotometer. A standard curve was prepared using 1,1,3,3-tetraethoxypropane with concentrations ranging from 0.3 to 10 μmol/L.

### 2.10. Assay of Catalase (CAT) Enzyme Activity

The CAT enzyme activity in plasma and liver tissue was quantified using a previously established method [32]. In brief, 20 µL of plasma was combined with 100 µL of substrate (65 µmol/mL hydrogen peroxide; H_2_O_2_) in 0.06 M sodium-potassium phosphate (NaPO_4_) buffer (pH 7.0) at 37 °C for 1 min, in a 96-well plate. The enzymatic reaction was halted by adding 100 µL of 32.4 mM ammonium molybdate (NH_46_)(Mo_7_O_24_·4H_2_O). The resulting yellowish molybdate and H_2_O_2_ complex were assessed at a wavelength of 405 nm. A standard curve was established using concentrations of bovine liver catalase ranging from 3.125 to 100 U/mL. The serum CAT activity was reported as U/mL.

### 2.11. Tumor Necrosis Factor-α (TNF-α) and Interleukin-6 (IL-6) Assay

Serum TNF-α and IL-6 concentrations were measured using commercial kits, Rat TNF alpha ELISA Kit (ab236712, Abcam, Cambridge, MA, USA) and Rat High Sensitive IL-6 (interleukin 6) ELISA Kit (RE3186RG, AFSBio, Wuhan, China), respectively. All protocols adhered completely to the manufacturer’s specifications.

### 2.12. Histological Examination

Liver histological examinations were performed as previously described [33]. The liver tissues were exercised and fixed in a 4% paraformaldehyde solution for 24 h before processing. Following fixation, standard processing procedures were applied, including embedding the tissues in paraffin and sectioning them at a thickness of 5 µm. To ensure accurate analysis and avoid duplicate counting, three sections, spaced at least 100 µm apart, were selected for histological examination. Hepatic morphologies were assessed using tissue sections stained with H&E. Images were captured using a DS-2Mv light microscope (Nikon, Tokyo, Japan), and 12 images were randomly chosen for quantification per sample. Each image was divided into quadrants to facilitate the grading of steatosis percentages. Hepatic steatosis was evaluated based on a percentage of fat accumulation in liver tissues as previously described [34]. The droplets were categorized based on their size: macrovesicular steatosis, which occupies a significant portion of the cytoplasm and may displace the nucleus; microvesicular steatosis, which is more evenly distributed throughout the cytoplasm without affecting nuclear position; and total steatosis, representing the sum of both macrovesicular and microvesicular steatosis.

### 2.13. Western Blot Analysis

The Western blot technique assessed the protein expression of insulin receptor substrate 1 (IRS-1) and protein kinase B (Akt) in the liver. Liver tissues were homogenized in ice-cold lysis buffer. Proteins were then separated by sodium dodecyl sulfate-polyacrylamide gel electrophoresis (SDS-PAGE), transferred to a PVDF membrane, and blocked with 5% skim milk in TBS containing 0.1% Tween 20 for one hour at 24 °C. Membranes were incubated overnight at 4 °C with mouse monoclonal antibodies IRS-1 (sc8038, 1:400) (Santa Cruz Biotechnology, Dallas, TX, USA) and Akt (9272, 1:1000) (Cell Signaling Technology, Danvers, MA, USA). The membranes were washed three times with TBST and then incubated with horseradish peroxidase-conjugated secondary antibodies for two hours at room temperature. β-actin (Santa Cruz Biotechnology, Santa Cruz, CA, USA) served as the loading control. The blots were developed using Amersham™ ECL™ Prime solution, and densitometric analysis was performed using ImageQuant™ LAS 4000 (GE Healthcare Life Science, Piscataway, NJ, USA). Band intensities were compared to β-actin, and the results were expressed as a percentage relative to the control group from the same gel.

### 2.14. Statistical Analysis

Data were analyzed using GraphPad Prism version 9 (GraphPad Software Inc., San Diego, CA, USA). The findings are expressed as mean values with a standard error of the mean (±S.E.M.). Statistical significances were assessed through a one-way analysis of variance (ANOVA) followed by Tukey’s post hoc test. All data were analyzed using GraphPad Prism software, and statistical significance was determined at a *p*-value < 0.05.

## 3. Results

### 3.1. Effect of Tangeretin on Body and Organ Weight

The initial body weight of each group before the start of the experiment was not significantly different. At the end of the 16-week experiment, the body weight of the metabolic syndrome rats was significantly greater than that of the control group (*p* < 0.05). In addition, liver weight, liver weight/body weight, visceral adipose tissue weight, visceral adipose tissue/body weight, epididymal adipose tissue weight, and epididymal adipose tissue/body weight of metabolic syndrome rats were significantly higher than in control rats. Treatment with tangeretin or metformin significantly reduced weight gain and visceral organ weight in the metabolic syndrome rats (*p* < 0.05) compared to the untreated group (Table 1).

### 3.2. Role of Tangeretin on Metabolic Parameters

**Figure 2:**
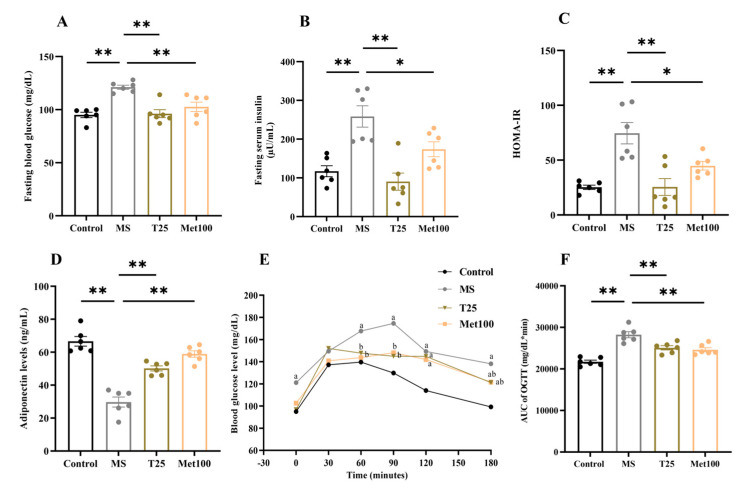
Effect of tangeretin on metabolic parameters including fasting blood glucose (**A**), fasting serum insulin (**B**), HOMA-IR index (**C**), adiponectin levels (**D**), blood glucose level of oral glucose tolerant test (OGTT) (**E**), and area under the curve (AUC) of OGTT (**F**). The data are presented as the mean ± S.E.M. (n = 6). * *p* < 0.05, ** *p* < 0.01, ^a^ *p* < 0.05 significant difference with the control group, ^b^ *p* < 0.05 significant difference with the MS group. MS: metabolic syndrome, T25: metabolic syndrome + tangeretin 25 mg/kg, Met100: metabolic syndrome + metformin 100 mg/kg.

Insulin resistance was observed in metabolic syndrome rats, with significant increases in fasting blood glucose (121.16 ± 1.95 mg/dL), fasting serum insulin (258.66 ± 27.26 µU/mL), and HOMA-IR (74.58 ± 9.72) and decreased adiponectin levels (29.72 ± 3.03 ng/mL) compared to normal rats (95.00 ± 2.59 mg/dL, 117.29 ± 14.16 µU/mL, 25.29 ± 1.95, and 66.62 ± 2.87 ng/mL) (*p* < 0.05). Tangeretin and metformin administration significantly improved insulin sensitivity by reducing fasting blood glucose (96.16 ± 3.79 mg/dL), (102.66 ± 2.59 mg/dL), fasting serum insulin (90.37 ± 22.26 µU/mL), (173.85 ± 19.18 µU/mL), and HOMA-IR index (25.50 ± 7.66), (44.83 ± 3.92), and adiponectin level (50.17 ± 1.55 ng/mL), (58.94 ± 1.95 ng/mL) in metabolic syndrome rats (*p* < 0.05) compared to the untreated group (Figure 2).

### 3.3. Role of Tangeretin on Lipid Profiles

Dyslipidemia was observed in metabolic syndrome rats, supported by a significant increase in serum TC, TG, and FFAs, along with a reduction in serum HDL-c compared to normal rats (*p* < 0.05). However, treatment with tangeretin and metformin significantly alleviated these lipid abnormalities (*p* < 0.05, Table 2).

### 3.4. Effect of Tangeretin on Liver Function

**Figure 3:**
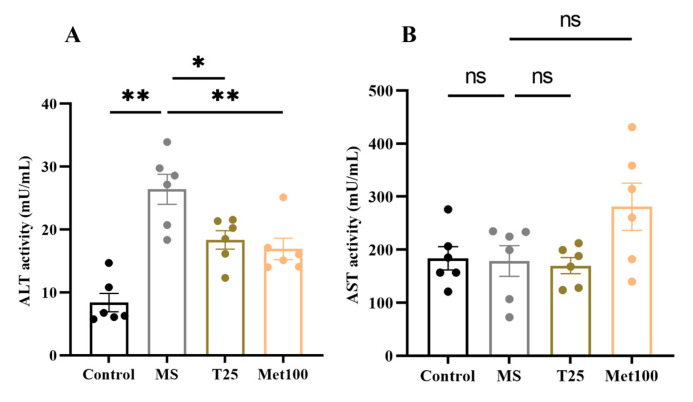
Effect of tangeretin on liver function, alanine aminotransferase (ALT) (**A**), aspartate aminotransferase (AST) (**B**). The data are presented as the mean ± S.E.M. (n = 6). * *p* < 0.05, ** *p* < 0.01. MS: metabolic syndrome, T25: metabolic syndrome + tangeretin 25 mg/kg, Met100: metabolic syndrome + metformin 100 mg/kg.

Serum ALT activity was significantly high (26.41 ± 2.38 mU/mL) in the metabolic syndrome rats compared to the control group (8.39 ± 1.47 mU/mL) (*p* < 0.05, Figure 3A). In contrast, supplementation with tangeretin and metformin significantly reduced ALT content (18.35 ± 1.46 mU/mL, 16.92 ± 1.70 mU/mL) compared to the untreated group (*p* < 0.05). However, serum AST was not different between the groups (Figure 3B).

### 3.5. Effect of Tangeretin on Liver Oxidative Stress in Metabolic Syndrome Rats

**Figure 4:**
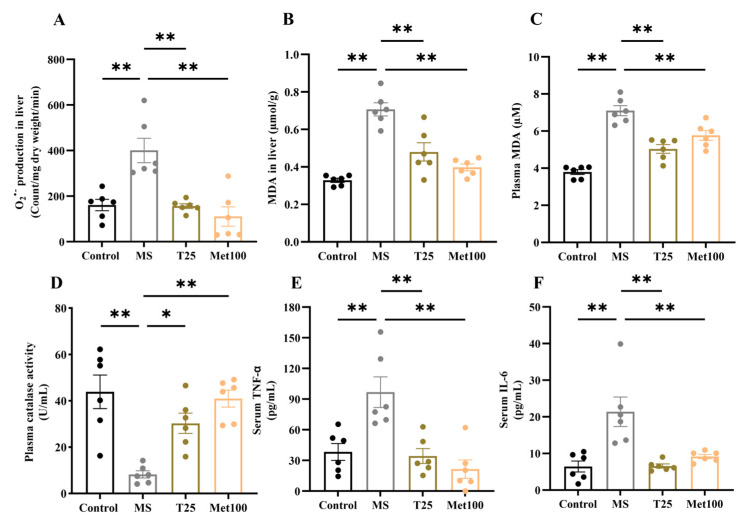
Effect of tangeretin on oxidative stress and inflammation in MS rats, O_2_^•−^ production level in liver tissue (**A**), MDA levels in liver tissue (**B**), MDA levels in plasma (**C**), catalase enzyme activity in plasma (**D**), serum TNF-α (**E**), and serum IL-6 (**F**). The data are presented as the mean ± S.E.M. (n = 6). * *p* < 0.05, ** *p* < 0.01. MS: metabolic syndrome, T25: metabolic syndrome + tangeretin 25 mg/kg, Met100: metabolic syndrome + metformin 100 mg/kg.

An imbalance of reactive oxygen species and an endogenous enzyme was found in a metabolic syndrome group supported by increased O_2_^•−^ production in liver tissues (400.88 ± 53.62 count/mg dry weight/min), MDA level in liver tissues (0.70 ± 0.03 µmol/g), plasma (7.10 ± 0.27 µM) and decreased CAT enzyme activity (8.22 ± 1.55 U/mL) in plasma compared to normal rats (161.32 ± 24.71 count/mg dry weight/min, 0.32 ± 0.01 µmol/g, 3.79 ± 0.13 µM), (43.86 ± 7.26 U/mL) (*p* < 0.05). Conversely, these parameters were alleviated in metabolic syndrome rats treated with tangeretin (156.63 ± 10.55 count/mg dry weight/min, 0.47 ± 0.04 µmol/g, 5.03 ± 0.23 µM, 30.27 ± 4.39 U/mL) and metformin (110.73 ± 42.25 count/mg dry weight/min, 0.39 ± 0.01 µmol/g, 5.76 ± 0.26 µM, 40.93 ± 3.64 U/mL) (*p* < 0.05) compared to untreated metabolic syndrome rats (Figure 4). Inflammation was present in metabolic syndrome rats, supported by high levels of serum TNF-α (96.88 ± 15.02 pg/mL) and IL-6 (21.38 ± 4.02 pg/mL) compared to control rats (TNF-α: 38.30 ± 8.25 pg/mL, 6.44 ± 1.50 pg/mL) (*p* < 0.05, Figure 4E,F). Tangeretin and metformin significantly reduced these inflammatory markers (TNF-α, (T25: 34.33 ± 7.28 pg/mL, Met100: 21.56 ± 8.98 pg/mL) and IL-6, (T25: 6.58 ± 0.55 pg/mL, Met100: 9.17 ± 0.58 pg/mL)) in this animal model (*p* < 0.05).

### 3.6. Effect of Tangeretin on Lipid Droplets Accumulation in Hepatocytes

**Figure 5:**
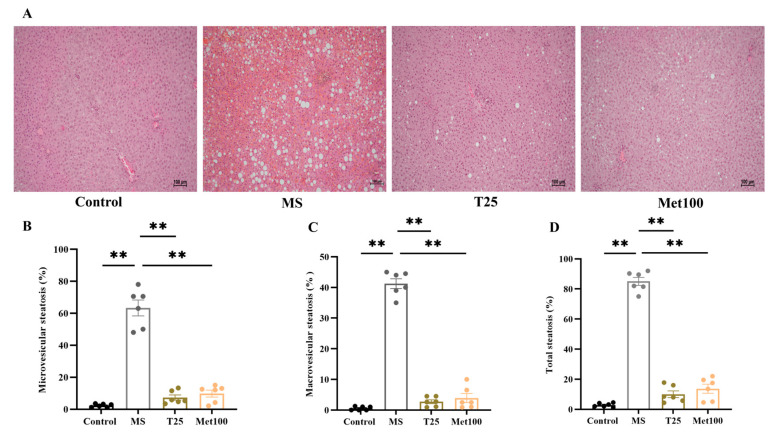
Effect of tangeretin on hepatic steatosis. Representative images of hepatocyte sections (magnification ×10, scale bar = 100 µm) stained with H&E (**A**). Microvesicular steatosis (**B**). Macrovesicular steatosis (**C**). Total steatosis (**D**). The data are presented as the mean ± S.E.M. (n = 6). ** *p* < 0.01. MS: metabolic syndrome, T25: metabolic syndrome + tangeretin 25 mg/kg, Met100: metabolic syndrome + metformin 100 mg/kg.

Histological evaluation of the liver stained by H&E showed significant accumulation of lipid droplets, or steatosis, in hepatocytes in the metabolic syndrome group (Figure 5A). Furthermore, significant increases in microvesicular steatosis (63.33 ± 4.93%), macrovesicular steatosis (41.25 ± 1.61%), and total steatosis (85.04 ± 2.69%) were also observed compared to the control group (2.30 ± 0.42%, 0.55 ± 0.25%), and (2.84 ± 0.53%, *p* < 0.05). Administration of tangeretin and metformin reduced microvesicular steatosis (7.34 ± 1.64%, 9.80 ± 2.16%), macrovesicular steatosis (2.72 ± 0.64%, 3.90 ± 1.47%), and total lipid steatosis (10.06 ± 2.24%, 13.70 ± 3.02%), compared to the untreated metabolic syndrome (*p* < 0.05, Figure 5B–D).

### 3.7. Effect of Tangeretin on IRS-1 and Akt Expression

**Figure 6:**
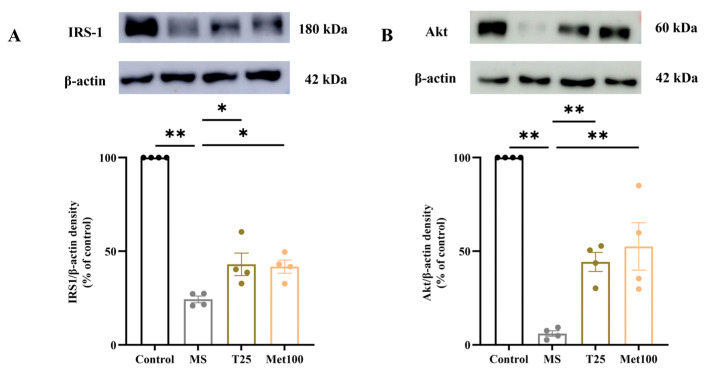
Effects of tangeretin on the expression of IRS-1 and Akt protein in liver tissue. IRS-1 protein expression (**A**) and Akt protein expression (**B**). The data are presented as the mean ± S.E.M. (n = 4). * *p* < 0.05, ** *p* < 0.01. MS: metabolic syndrome, T25: metabolic syndrome + tangeretin 25 mg/kg, Met100: metabolic syndrome + metformin 100 mg/kg.

IRS-1 (24.33 ± 1.76% of control) and Akt (6.07 ± 1.46% of control) protein expression in liver tissue were downregulated in metabolic syndrome rats compared to normal rats (100 ± 0.00% of control), (100 ± 0.00% of control) (*p* < 0.05). Supplementation with tangeretin (43.04 ± 5.98% of control), (44.30 ± 5.09% of control), and metformin (41.80 ± 3.48% of control), (52.53 ± 12.66% of control) recovered IRS-1 and Akt protein expression compared to untreated metabolic syndrome rats (*p* < 0.05, Figure 6).

## 4. Discussion

An animal model of metabolic syndrome associated with fatty liver was established in this study using an HFD plus fructose solution. HFD-induced metabolic syndrome and MAFLD rats showed obesity, hyperglycemia, insulin resistance, and dyslipidemia. These signs of metabolic syndrome were alleviated by tangeretin treatment. Tangeretin also resolved the low concentration of adiponectin, the impairment of liver function, and an imbalance of reactive oxygen species and endogenous antioxidant defense enzymes observed in the MAFLD group. The elevated concentrations of cytokines, TNF-α, and IL-6 caused by HFD were ameliorated by tangeretin treatment. The histology analysis indicated lipid droplet formation in the livers of rats given an HFD. Tangeretin reduced microvesicular and macrovesicular steatosis in rats fed HFD plus fructose solution. The insulin receptor pathway, IRS-1/Akt protein expression, was decreased in MAFLD rat livers but restored with tangeretin therapy. Metformin was assigned as a positive control drug, and it alleviated signs of metabolic syndrome and MAFLD in rats that received the HFD plus fructose solution.

Excessive consumption of high-energy foods rich in fat and fructose can lead to obesity, increased visceral and epididymal fat, insulin insensitivity, hyperglycemia, and dyslipidemia, all of which are Mets. This study demonstrated that rats subjected to HFD combined with a fructose solution developed metabolic syndrome. Our results were consistent with a previous study that established animal models of Mets by feeding HFD and fructose [35]. It has been known that HFD enhances visceral fat storage and FFAs, which trigger insulin resistance and high blood glucose [36]. Furthermore, fructose metabolism differs from glucose in that fructolysis is not regulated by fructose metabolites, ATP, or trioses-phosphate. As a result, high fructose intake can easily be converted into FFAs, TG, and LDL-c, all of which contribute to obesity and metabolic syndrome [37,38]. Fructose also inhibits hepatic fatty acid oxidation and HDL-c production, which contributes to visceral obesity and increased body mass. In addition, fructose diets impair insulin sensitivity by lipogenesis, resulting in insulin resistance and, eventually, type 2 diabetes [39]. In this work, we also discovered reduced levels of adiponectin in HFD rats, which may enhance insulin resistance in this animal model. Our findings were corroborated by the previous discovery that adiponectin concentrations were reduced in rats with diet-induced insulin resistance [40]. Direct evidence showed that treatment with adiponectin normalized insulin sensitivity in the liver and muscle in HFD-fed mice [41]. We observed that tangeretin treatment mitigated metabolic syndrome and raised adiponectin levels in rats fed with HFD and fructose. The exact mechanism by which tangeretin influences metabolic syndrome remains uncertain. Nevertheless, our findings aligned with prior research. For example, tangeretin has been shown to diminish insulin resistance in rats subjected to HFD by mitigating inflammation [42]. The effect of tangeretin on insulin sensitivity in this study was linked to its capacity to raise adiponectin concentration. In obese C57BL/6J mice that received an HFD, tangeretin reduced blood glucose, insulin resistance, and visceral fat, attributed to its anti-inflammatory effects [43]. Tangeretin alleviates dyslipidemia by lowering cholesterol and triacylglycerol in hamsters with diet-induced dyslipidemia [44].

This study revealed that rats exhibiting symptoms of metabolic syndrome developed fatty livers. The liver enzyme, ALT, was elevated, although AST remained unchanged in HFD rats. The imbalance of hepatic antioxidant enzymes and oxidative stress indicators was identified in high-fat diet rats. Histopathology revealed lipid droplet accumulation, including microvesicular and macrovesicular steatosis, in the liver of HFD rats. It is well known that rats treated with HFD plus fructose can develop fatty liver as MAFLD [45]. Our findings were corroborated by previous reports indicating that fatty liver caused by HFD is associated with elevated liver weight, blood ALT levels, and oxidative stress [10,46]. We found the ALT raised while AST did not change, proposing liver damage in HFD rats. It has been suggested that the major biomarkers of hepatic function are liver enzymes, AST and ALT, with AST detectable in the liver as well as other tissues such as erythrocytes, skeletal muscle, and the heart. Thus, ALT is a more specific indication of liver injury than AST. Moreover, signs of metabolic syndrome such as insensitivity, high blood glucose, and dyslipidemia can induce oxidative stress and mediate hepatic damage [6,15]. In HFD rats, tangeretin therapy for four weeks alleviated MAFLD. This effect was accompanied by reductions in liver weight, serum ALT, and liver oxidative stress. The alleviative effect of tangeretin on hepatic steatosis in HFD-induced NAFLD mice via enhancing hepatic antioxidant enzymes and lowering oxidative stress markers has been reported [47]. Tangeretin-reduced oxidative stress in the present study may involve at least two mechanisms; a direct effect, tangeretin exhibits its antioxidant capacity [20], and an indirect impact, tangeretin improves insulin sensitivity and dyslipidemia [43] and then reduces oxidative stress. This study demonstrated that HFD rats displayed inflammation, as seen by increased serum levels of TNF-α and IL-6. As a result, inflammation and oxidative stress in MAFLD rats may indicate that hepatic steatosis is developing into nonalcoholic steatohepatitis (NASH) [48]. We found that serum concentrations of TNF-α and IL-6 of HFD rats treated with tangeretin were not different from control rats. Tangeretin exhibits potent anti-inflammatory properties by reducing proinflammatory cytokines, TNF-α and IL-6, in rats exposed to cerebral ischemia [49] and murine macrophage RAW264.7 cells [50].

We further explored the hepatic protein expression of the insulin signaling pathway to confirm the involvement of insulin resistance in fatty liver. Hepatic insulin resistance was seen in HFD rats supported by downregulation of IRS-1/Akt protein expression. However, in HFD rats treated with tangeretin, these protein expressions were not different from the control rats. Reduction of the insulin signaling pathway impaired hemostasis, lipid metabolism, and liver function [51,52,53]. Our findings supported the earlier work [14] showing hepatic insulin resistance is associated with excessive lipid accumulation in the liver and the advancement of MAFLD. According to this study, tangeretin improved the insulin signaling pathway that could enhance hepatic hemostasis. We acknowledge that mRNA expression analysis could provide additional insights into the regulation of the IRS/Akt pathway. However, whether tangeretin modulates gene expression directly or primarily through post-translational modifications remains unexplored. Future research using quantitative RT-PCR could provide further insights into how tangeretin affects gene transcription.

Metformin, a biguanide, is used for type II diabetes treatment as it potentially reduces blood glucose [54]. In this investigation, metformin was assigned as a positive control agent. It reduced metabolic syndrome, oxidative stress, and fatty liver in HFD-fed rats. There is substantial evidence that metformin reduces blood glucose, serum insulin, LDL-c, triglycerides, and liver damage in rats fed with HFD [55]. Metformin has been reported to have an antioxidant capacity that mitigates HFD-induced NAFLD and dyslipidemia in rats [56]. The mechanism’s action of metformin involves the activation of AMPK to suppress hepatic gluconeogenesis and promote GLUT4-mediated glucose transport [57]. Similarly, tangeretin activates AMPK to enhance glucose uptake and GLU4 translocation in C2C12 myotube and heart tissue from obese mice [58]. A notable distinction between these two therapies was the dosages of tangeretin (25 mg/kg) and metformin (100 mg/kg). In comparison to metformin at the same concentration, tangeretin had greater efficacy. This suggests that tangeretin may exert potent metabolic benefits, potentially through mechanisms beyond those of metformin. Our findings align with previous research, which reported that tangeretin at a dose of 25 mg/kg improved insulin sensitivity and glucose metabolism in mice [28]. Moreover, metformin at doses of 100 mg/kg decreased fasting glucose and insulin resistance in mice fed with HFD [59].

## 5. Conclusions

In conclusion, this study showed that tangeretin ameliorated hyperglycemia, dyslipidemia, insulin resistance, and fatty liver in HFD-fed rats. This beneficial effect of tangeretin on MAFLD involves its antioxidant and anti-inflammatory capacities and enhancing IRS1/Akt protein expression. These data suggest that tangeretin may serve as a viable therapeutic agent for managing metabolic disorders associated with insulin resistance and oxidative stress, including MAFLD. Future research may concentrate on clinical transformation to enhance its efficacy in MAFLD management.

## Figures and Tables

**Table 1:** Effect of tangeretin on body and organ weight in metabolic syndrome rats.

Parameters	Control	MS	T25	Met100
Initial BW (g)	205.66 ± 3.67	204.88 ± 3.83	204.36 ± 4.05	199.53 ± 4.19
Final BW (g)	605.70 ± 10.69	947.12 ± 33.04 ^a^	824.00 ± 33.40 ^ab^	810.91 ± 35.00 ^ab^
Food intake (g/day)	29.86 ± 0.37	26.93 ± 0.74	27.44 ± 0.78	27.48 ± 0.95
Calorie intake (Kcal/day)	111.77 ± 1.41	144.09 ± 3.43 ^a^	145.26 ± 3.68 ^a^	147.37 ± 4.39 ^a^
Liver weight (g)	14.73 ± 0.47	26.62 ± 1.13 ^a^	20.83 ± 1.18 ^b^	21.56 ± 1.19 ^b^
Liver/BW ratio (%)	2.32 ± 0.05	2.83 ± 0.06 ^a^	2.47 ± 0.07 ^b^	2.54 ± 0.05 ^b^
Visceral adipose tissue weight (g)	15.27 ± 1.05	71.98 ± 4.39 ^a^	43.98 ± 5.7 ^ab^	36.55 ± 7.73 ^ab^
Visceral adipose tissue/BW (%)	2.51 ± 0.15	7.70 ± 0.35 ^a^	5.14 ± 0.34 ^ab^	4.65 ± 0.82 ^ab^
Epididymal adipose tissue weight (g)	10.86 ± 0.22	26.44 ± 20.21 ^a^	20.21 ± 1.30 ^ab^	20.45 ± 2.73 ^ab^
Epididymal adipose tissue/BW (%)	1.77 ± 0.04	3.08 ± 00.8 ^a^	2.40 ± 0.02 ^ab^	2.58 ± 0.25 ^ab^

The data are presented as the mean ± S.E.M. (n = 6). ^a^ *p* < 0.05 significant difference with the control group, ^b^ *p* < 0.05 significant difference with the MS group. BW: body weight, MS: metabolic syndrome, T25: metabolic syndrome + tangeretin 25 mg/kg, Met100: metabolic syndrome + metformin 100 mg/kg.

**Table 2:** Tangeretin improved lipid profiles in metabolic syndrome rats.

Parameters	Control	MS	T25	Met100
Serum total cholesterol (mmol/L)	17.97 ± 1.41	38.42 ± 3.70 ^a^	19.13 ± 4.67 ^b^	18.66 ± 9.15 ^b^
Serum triglyceride (mmol/L)	0.99 ± 0.35	5.08 ± 0.32 ^a^	1.05 ± 0.19 ^b^	3.58 ± 0.33 ^ab^
Serum HDL-c (mmol/L)	38.88 ± 7.10	14.58 ± 1.64 ^a^	41.11 ± 5.45 ^b^	51.38 ± 3.52 ^b^
Serum free fatty acid (μM)	201.81 ± 2.36	619.91± 60.21 ^a^	202.73 ± 49.88 ^b^	183.56 ± 29.27 ^b^

The data are presented as the mean ± S.E.M. (n = 6). ^a^ *p* < 0.05 significant difference with the control group, ^b^ *p* < 0.05 significant difference with the MS group. MS: metabolic syndrome, T25: metabolic syndrome + tangeretin 25 mg/kg, Met100: metabolic syndrome + metformin 100 mg/kg.

## Data Availability

All data generated for the manuscript are included in the study and are available upon request.

## Abbreviations

The following abbreviations are used in this manuscript:

Akt	Protein Kinase B
ALT	Alanine transaminase
AST	Aspartate transaminase
ATP	Adenosine Triphosphate
AUC	Area under the curve
FFAs	Free fatty acid
IL-6	Interleukin 6
HDL-c	High-density lipoprotein cholesterol
HFD	High-fat diet
HOMA-IR	Homeostatic model assessment of insulin resistance
IRS-1	Insulin receptor substrate 1
LDL-c	Low-density lipoprotein cholesterol
MAFLD	Metabolic dysfunction-associated fatty liver disease
MDA	Malondialdehyde
Met	Metformin
Mets	Metabolic syndrome
NAFLD	Non-alcoholic fatty liver disease
NF-κB	Nuclear factor kappa-light-chain-enhancer of activated B cells
Nrf2	Nuclear factor erythroid-derived 2-like 2
O_2_^•−^	Superoxide anion
OGTT	Oral glucose tolerance test
PI3K	Phosphoinositide 3-kinase
TC	Total cholesterol
TG	Triglycerides
TNF-α	Tumor necrosis factor-α
